# PAVS: A New Privacy-Preserving Data Aggregation Scheme for Vehicle Sensing Systems

**DOI:** 10.3390/s17030500

**Published:** 2017-03-03

**Authors:** Chang Xu, Rongxing Lu, Huaxiong Wang, Liehuang Zhu, Cheng Huang

**Affiliations:** 1 School of Computer Science and Technology, Beijing Institute of Technology, Beijing 100081, China; xuchang@bit.edu.cn (C.X.); liehuangz@bit.edu.cn (L.Z.); 2 Division of Mathematical Sciences, School of Physical & Mathematical Sciences, Nanyang Technological University, Singapore 639798, Singapore; hxwang@ntu.edu.sg; 3 Faculty of Computer Science, University of New Brunswick, Fredericton, NB E3B 5A3, Canada; 4 Department of Electrical and Computer Engineering, University of Waterloo, Waterloo, ON N2L 3G1, Canada; c225huan@uwaterloo.ca

**Keywords:** vehicle sensing, data aggregation, privacy-preserving aggregation, privacy-preserving data statistics

## Abstract

Air pollution has become one of the most pressing environmental issues in recent years. According to a World Health Organization (WHO) report, air pollution has led to the deaths of millions of people worldwide. Accordingly, expensive and complex air-monitoring instruments have been exploited to measure air pollution. Comparatively, a vehicle sensing system (VSS), as it can be effectively used for many purposes and can bring huge financial benefits in reducing high maintenance and repair costs, has received considerable attention. However, the privacy issues of VSS including vehicles’ location privacy have not been well addressed. Therefore, in this paper, we propose a new privacy-preserving data aggregation scheme, called PAVS, for VSS. Specifically, PAVS combines privacy-preserving classification and privacy-preserving statistics on both the mean E(·) and variance Var(·), which makes VSS more promising, as, with minimal privacy leakage, more vehicles are willing to participate in sensing. Detailed analysis shows that the proposed PAVS can achieve the properties of privacy preservation, data accuracy and scalability. In addition, the performance evaluations via extensive simulations also demonstrate its efficiency.

## 1. Introduction

Air pollution has become a major environmental risk factor for ill health and death. Epidemiological studies have showed that long-term exposure to PM 2.5 can cause heart disease, stroke, and lung cancer, etc. [1]. In order to attain air pollution monitoring, a series of solutions have been proposed [2,3,4]. However, traditional monitoring equipment is usually stationary, complex, and expensive due to the high cost of construction and maintenance. In contrast, vehicle sensing systems (VSS) have attracted more attention, since vehicles can be equipped with various kinds of sensors that can achieve collection and concentration measurements of a range of pollutants [5]. Specifically, the sensing data are firstly collected by vehicle sensors [6], transferred to roadside units (RSUs) by vehicle wireless transmitters via vehicular ad hoc networks (VANET) [7,8], and then relayed to remote servers by RSUs.

In recent years, VSS has been regarded as a new tool to monitor gas concentration and has attracted more and more attention. Lee et al. [9] pointed out that VSS can be used to collect data when criminals spread poisonous chemicals in flight. Hu et al. [10] proposed exploiting VSS to achieve carbon dioxide monitoring. Specifically, the vehicles can be taxis or buses that collect carbon dioxide concentration and periodically report their locations and concentration. In addition, VSS can also be used for traffic monitoring. According to [11], average speed or traffic density should be collected by departments of transportation in the USA for traffic monitoring purposes. Though the traditional technologies can help collect these data, these technologies suffer high maintenance and repair costs.

Designing a VSS refers to numerous problems, e.g., how to increase the sensing coverage. Accordingly, some excellent solutions have been proposed to enhance sensing coverage and reduce detection time in vehicular sensor networks [12,13,14,15,16]. Moveover, a series of aggregation schemes have been proposed [17,18,19,20]. However, these aggregation schemes are only used to reduce the overhead of transmitted sensing data. Specifically, all of the aforementioned studies did not consider how to hide the real identities and location information of vehicles.

Therefore, how to achieve privacy preservation [21,22,23,24,25,26] becomes one of the most critical problems for VSS. In VSS, after the sensing data are analyzed, the statistical data e.g., the mean E(·) and variance Var(·) will probably be published in public [10]. In this case, we find there exists an attack (we call it a *sensing data link attack*), in which attackers may learn the vehicle’s previous location information by linking the data collected by vehicles with the published statistical data. This kind of “*sensing data link attack*” may breach the location privacy [27] of vehicles, since location privacy of vehicles may include the drivers’ living places, companies, and the amusement places to which they usually go, etc. [28,29,30]. Moreover, leakage of privacy is possible to produce negative effects [31]. One of the possible solutions to resist the *sensing data link attack* is to encrypt data and transmit ciphertexts to RSUs. However, it causes some problems in aggregation of encrypted data, e.g., how to classify the ciphertexts on the RSU side according to where the data are collected, and how to efficiently compute statistical data from aggregation results on the service provider side.

Aiming at the above challenges, in this paper, we propose a new privacy-preserving data aggregation scheme for VSS, called PAVS. To the best of our knowledge, it is the first work to address this “sensing data link attack” and present a privacy-preserving data aggregation scheme to compute both the mean E(·) and variance Var(·) of sensing data for VSS. Specifically, the main contributions of this paper are fourfold:

- We propose new privacy-preserving data classification and privacy-preserving aggregation algorithms, so that service providers can efficiently compute the mean E(·) and variance Var(·) from aggregation results. In addition, the proposed PAVS captures data accuracy, i.e., the E(·), and Var(·) computed from each aggregation data map to a specific area and time period.
- The proposed PAVS holds privacy-preserving property. Specifically, it can resist *sensing data link attack*. After executing PAVS, RSUs cannot get any valuable information of vehicles including vehicles’ previous location information and real identities.
- The PAVS scheme achieves scalability. If a service provider holds the aggregation results of areas $Area_{1},...,Area_{t}$, respectively, it can further compute the statistical data of a larger area that consists of $Area_{1},...,Area_{t}$ by performing aggregation operations, without re-executing the whole PAVS scheme.
- To demonstrate the utility and validate the efficiency of the proposed PAVS, we theoretically analyze the performance of PAVS in terms of computational cost, communication cost and storage cost. Additionally, we develop a Java simulator to simulate the computational cost on the vehicle side, RSU side and service provider side. The experiment results show that the proposed PAVS is efficient at the three sides.

The rest of the paper is organized as follows. In Section 2, we formalize the system model, security model and identify the design goal. In Section 3, we introduce bilinear pairing, related complexity assumptions, and properties of group $Z_{p^{2}}^{*}$ as preliminaries. The proposed PAVS scheme is described in Section 4, followed by the security analysis in Section 5 and the performance evaluation in Section 6. The related work is given in Section 7, and we conclude this work in Section 8.

## 2. Models and Design Goal

In this section, we formulate the system model, the security model and identify the design goal.

### 2.1. System Model

**Figure 1:**
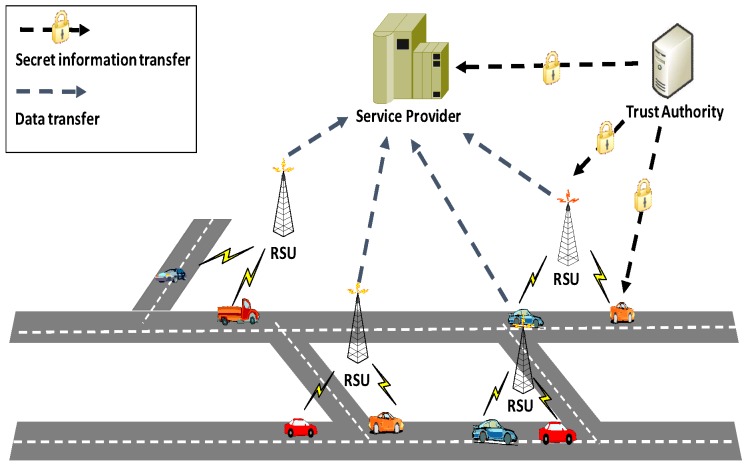
System model.

In VSS, the sensing data are collected by vehicles, transmitted to RSUs, and then transferred to the service providers [6]. In our system model, the service provider further deals with the data and publishes the results of statistical analysis in public. Our model consists of four kinds of entities: trusted authority, service provider, RSUs, and vehicles (as shown in Figure 1).

- Trusted Authority (TA): TA’s duty is to manage and distribute key materials to service providers, RSUs, and vehicles in the system.
- Service Provider (SP): SP deals with each aggregation result received from an RSU and gets E(·) and Var(·) for each area.
- RSUs: Each RSU serves as a message aggregator role in the system. An RSU aggregates the messages sent from vehicles and forwards the aggregation results to SP. Before executing aggregation operations, RSUs will first classify the messages according to where and when the sensing data are collected.
- Vehicles: Each vehicle is equipped with sensor devices. Vehicles can then collect data in different areas and transfer messages to RSUs in batch.

### 2.2. Security Model

In our security model, TA and SP are fully trusted. For RSUs, on one hand, RSUs will follow the designated protocol specification. On the other hand, RSUs are curious and may try to disclose vehicles’ privacy information. Specifically, RSUs can get all the messages transferred in the protocol. After RSUs get all the messages, RSUs may try decrypt the ciphertext to get sensing data and launch sensing data link attacks by linking the messages sent by vehicles and the statistical results.

We will show that PAVS can resist *sensing data link attack* by introducing two levels of privacy: basic privacy and full privacy. Specifically, we will prove that PAVS holds full privacy to demonstrate that RSUs cannot link the messages sent by vehicles and the statistical results published by SP. Note that the collision of RSUs and SP is beyond the scope of this paper.

**Definition 1** (Basic Privacy). *When a run of the protocol is completed, RSUs cannot obtain vehicles’ real identity information by communicating with vehicles.*

**Definition 2** (Full Privacy). When a run of the protocol is completed, RSUs cannot obtain vehicles’ real identity information and any other information with vehicles.

### 2.3. Design Goal

Under the aforementioned system model and security model, our design goal is to propose an efficient privacy-preserving data aggregation scheme for VSS, so that SP can obtain more abundant information from each aggregation result without vehicles’ privacy leakage. Particularly, the following four objectives should be captured:

- Privacy preservation. The privacy information of vehicles including previous location information and the real identities of vehicles should be protected.
- Accuracy. The mean E(·) and variance Var(·) computed by each aggregation result should map to a specific area and time period. Additionally, aggregation results should be generated by real RSUs, and all the sensing data should be collected by registered vehicles.
- Scalability. If SP has held the aggregation results for some small areas, E(·) and Var(·) for a larger area which consists of theses small areas should be efficiently computed without re-executing the whole scheme.
- Efficiency. The computation on the vehicle side, the RSU side and the SP side should be efficient.

## 3. Preliminaries

In this section, we will introduce bilinear pairing, related complexity assumptions, and properties of group $Z_{p^{2}}^{*}$ that will serve as the basis of our scheme.

### 3.1. Bilinear Pairing and Complexity Assumptions

Let G and $G_{T}$ be two multiplicative groups of order *q* for some large prime *q*, and *g* be a generator of G. A bilinear map $\hat{e}:G\times G\to G_{T}$, which satisfies the following properties:

- Bilinearity: $\hat{e}\left(g^{a},g^{b}\right)=\hat{e}{\left(g,g\right)}^{ab}$ for all $a,b\in Z_{q}^{*}$.
- Non-degeneracy: $\hat{e}\left(g,g\right)\neq 1$.
- Computability: $\hat{e}\left(x,y\right)$ can be computed efficiently.

**Definition 3** (Bilinear Generator). A bilinear parameter generator Gen is a probability algorithm that takes a security parameter κ as input and outputs a 5-tuple $\left(q,g,G,G_{T},\hat{e}\right)$, where q is a κ-bit prime number, (G,×) and $(\left(G_{T},\times \right)$ are two groups with the same order q, g∈G is a generator, and $\hat{e}:G\times G\to G_{T}$ is an admissible bilinear map.

**Definition 4** (Decisional Bilinear Diffie–Hellman (DBDH) Assumption). Let $\left(q,g,G,G_{T},\hat{e}\right)$ be the output of the bilinear parameter generator. Given $g,g^{a},g^{b},g^{c}\in G$ and $R\in G_{T},$ where a,b,c are random elements in $Z_{q}^{*}$, R is a random element in $G_{T}$. We say an algorithm B that outputs l∈{0,1} has advantage ε in solving the DBDH problem in G if

$$|\mathrm{Pr}\left[B\left(g,g^{a},g^{b},g^{c},\hat{e}{\left(g,g\right)}^{abc}\right)=0\right]-\mathrm{Pr}\left[B\left(g,g^{a},g^{b},g^{c},R\right)=0\right]|\geq \varepsilon .$$

### 3.2. Properties of Group $Z_{p^{2}}^{*}$

Given the security parameter *λ*, we choose a safe prime $p=2p^{\prime }+1$, where |p|=λ and $p^{\prime }$ is also a prime. Then, we can calculate the Euler’s totient function $\phi (p^{2})$ as $\phi \left(p^{2}\right)=p^{2}\left(1-1/p\right)=p\left(p-1\right)=2pp^{\prime }$. That is, the order of $Z_{p^{2}}^{*}$ is $2pp^{\prime }$. Let $x\in Z_{p}^{*}.$ According to Fermat’s Little Theorem, we have $x^{p-1}\equiv 1\;\mathrm{mod}\;p$. Thus, for some integer *k*, the equality $x^{p-1}=1+k\cdot p$ holds. Furthermore, we obtain

xp(p−1)=(1+k×p)p=1+∑i=1ppi(k×p)i=1modp2.

Let y=p+1. When k=1, we obtain

yp=(p+1)p=1+∑i=1ppipi=1modp2.

Thus, we get the following properties of group $Z_{p^{2}}^{*}$:

1 For any $x\in Z_{p}^{*}$, we have $x^{p(p-1)}=1\;\mathrm{mod}\;p^{2}$; and
2 for any y=p+1, the equality $y^{p}=1\;\mathrm{mod}\;p^{2}$ holds.

## 4. Proposed PAVS Scheme

In this section, we present our PAVS scheme, which mainly consists of the following parts: **System Initialization**, **Data Collection** at the vehicle, **Data Aggregation** at RSU, and **Statistical Analysis** at SP.

### 4.1. Overview

In the **System Initialization** phase, TA will mainly execute the Parameter Generation algorithm to generate public parameters and the Key Generation algorithm to generate key materials to vehicles, RSUs and SP.

In the **Data Collection** phase, the vehicles will encrypt the sensing data by performing a Data Encryption algorithm and sign the ciphertexts by running a Message Signing algorithm. After that, the vehicles will send the messages to RSUs.

In the **Data Aggregation** phase, RSUs will classify the messages according to where and when the sensing data are collected, and aggregate the data that are collected in the same area and the same time period. Then, RSUs send the aggregation results to SP.

In the **Statistical Analysis** phase, SP will decrypt the aggregation results and get the mean E(·) and variance Var(·) for each area.

In the vehicle sensing system, the sensing data are firstly collected by vehicle sensors, transferred to RSUs by vehicle wireless transmitters via VANET, and then relayed to remote servers by RSUs. As the reviewer mentioned, the data may not be able to arrive at the data aggregation at the same time; therefore, time stamps are included in PAVS. Thus, RSUs classify the messages according to the time stamps and the area where the data are sensed. That is, only the data with the same time stamp and collected in the same area will be aggregated together. Finally, SP computes the statistic data, i.e., the E(), and Var() from each aggregation data map to a specific area and time stamp.

### 4.2. System Initialization

This phase is mainly comprised of the ***Parameter Generation*** algorithm, the ***Key Generation*** algorithm, and the ***List Generation*** algorithm.

*Parameter Generation (PG):* On input security parameter *λ*, TA publishes system parameters

$$(q,G,G_{T},\hat{e},g,p,p^{\prime },\eta ,H,H_{1},H_{2}),$$

where $p=2p^{\prime }+1$ is a safe prime, |p|=λ, $p^{\prime }$ is a large prime; $\eta \in Z_{p}^{*}$ is a generator of $Z_{p^{2}}^{*}$; and $\left(q,g,G,G_{T},\hat{e}\right)$ is the output of the bilinear parameter generator. $H:{\left\{0,1\right\}}^{*}\to Z_{q}^{*},$ $H_{1}:{\left\{0,1\right\}}^{*}\to G,$ and $H_{2}:{\left\{0,1\right\}}^{*}\to Z_{p}^{*}$ are all cryptographic hash functions.

*Key Generation (KG)*: On input system parameters, TA generates its secret key $s_{0},$ its master private key s, area key $k_{0},$ and public parameter $P_{pub},$ where $s_{0},s,k_{0}\in Z_{q}^{*},$ and $P_{pub}=g^{s}$. Then, the following steps are executed:

***1***: TA computes private key $S_{L_{j}}$ for each RSU $R_{j},$ j∈{1,...,α}, where $S_{L_{j}}=H_{1}(L_{j}\left|\right|R_{j}{)}^{s},$ $R_{j}$ is the label of an RSU, and $L_{j}$ is the location of $R_{j}$.
***2***: TA generates pseudo-identity $PID_{i}$ for vehicle $V_{i},$ i∈{1,...,β}, where $PID_{i}=AES_{s_{0}}(v_{i}\left|\right|r_{i}),$ $v_{i}$ is the real identity of $V_{i},$ $r_{i}$ is randomly chosen in $Z_{q}^{*},$ AES is the symmetric encryption algorithm, and $s_{0}$ is used to generate the symmetric encryption key.
***3***: After TA authenticates $V_{i}$’s real identity, TA generates $V_{i}$’s private key $s_{i}\in Z_{q}^{*},$ computes $V_{i}$’s public key $g^{s_{i}}$ and authority key $g^{s_{i}r_{i}}$.
***4***: TA transfers $s_{i}$, $k_{0}$ and $PID_{i}$ to $V_{i},$ $S_{L_{j}}$ to $R_{j},$ and sends $k_{0}$, {$PID_{1},...,PID_{\beta }$} and {$g^{s_{1}r_{1}},...,g^{s_{\beta }r_{\beta }}$} to SP.

*List Generation (LG)*: TA generates the vehicles’ public key list (as shown in Table 1, the area list (as shown in Table 2), the RSU private key list (as shown in Table 3), the random value lists (as shown in Table 4 R-value list-1 and Table 5 R-value list-2), and the vehicle authority key list (as shown in Table 6 A-key list). The vehicles’ Public key list, Area list, and R-value list-2 are public, the A-key list is maintained by TA and SP secretly, and the RSU private key list and R-value list-1 are kept by TA secretly.

**Remark** **1.** *The communications between TA and each vehicle, between TA and SP, between TA and each RSU are all via private and authenticated channels. TA’s secret key $s_{0}$ is used to generate vehicles’ PIDs. TA uses its master private key s to generate RSUs’ private keys. The area key $k_{0}$ is also known by vehicles and SP, and SP utilizes $k_{0}$ to recover the area. By using R-value list-1, TA can recover the real identity $v_{i}$ according to $PID_{i}$. R-value list-2 is used by vehicles to encrypt sensing data. A-key list is utilized by SP to compute E(·) and Var(·) .*

### 4.3. Data Collection at Vehicle

After the vehicle $V_{i}$ collects sensing data, $V_{i}$ executes the following ***Data Encryption*** algorithm, ***Message Generation*** algorithm and ***Message Signing*** algorithms.

*Data Encryption (DE)*: Assume that $V_{i}$ collects $m_{i1},...,m_{i\theta_{i}}$ in $Area_{1},...,Area_{\theta_{i}}$, respectively, during the same time period. Let $m_{ir}\in \left\{0,1,2,\left\lfloor \frac{p}{\beta +1}\right\rfloor \right\},r\in \left[1,\theta_{i}\right]$ $\theta_{i}\leq t$, where *t* is the number of areas and *β* is the number of the registered vehicles in the system. In order that SP can compute E(·) and Var(·) of the sensing data of $Area_{r}$, $V_{i}$ encrypts $m_{ir}$ as follows:

$$C_{ir}={\left(p+1\right)}^{{m_{ir}}^{2}}\times \eta^{m_{ir}}\times H_{2}\left(\hat{e}{\left(H_{1}\left(T_{i},PID_{i}\right),g^{r_{i}}\right)}^{s_{i}}\right)\;\mathrm{mod}\;p^{2},$$

where $T_{i}$ is the time stamp.

**Remark** **2.** Note that the data may not be able to arrive at the data aggregation at the same time; therefore, time stamps are included in PAVS. Thus, RSUs classify the messages according to the time stamps and the area where the data are sensed. That is, only the data with the same time stamp and collected in the same area will be aggregated together. The unit of time stamp is set by TA. In real life, the unit of time stamp can be an hour or half an hour. For simplicity, the time stamp is denoted as $T_{i}$. That is, the subscript of T is denoted as i. In fact, the subscript can be set as any variable, since the time stamp is not related to the identities of the vehicles.

*Message Generation (MG)*: The messages sent from vehicle $V_{i}$ should include the PID of $V_{i}$, so that RSUs can recover the public key from the public key list to verify the signature generated by $V_{i}$. $V_{i}$ generates the message $M_{i}=\left(M_{i1},M_{i2},...,M_{i\theta_{i}},T_{i},PID_{i}\right),$ where

$$\left\{\begin{array}{ccc} M_{i1} & = & <H_{1}(Area_{1}\left|\right|T_{i}\left|\right|k_{0}{)}^{a_{i1}},g^{a_{i1}},C_{i1}>\\ M_{i2} & = & <H_{1}(Area_{2}\left|\right|T_{i}\left|\right|k_{0}{)}^{a_{i2}},g^{a_{i2}},C_{i2}>\\ ... & = & ...\\ M_{i\theta_{i}} & = & <H_{1}(Area_{\theta_{i}}\left|\right|T_{i}\left|\right|k_{0}{)}^{a_{i\theta_{i}}},g^{a_{i\theta_{i}}},C_{i\theta_{i}}>. \end{array}\right.$$

In addition, $a_{ir}$ is randomly chosen in $Z_{q}^{*},$ $r\in [1,\theta_{i}],$ and $Area_{r}$ is the area where $m_{ir}$ is collected. Here, $M_{ir}$ includes two messages $H_{1}(Area_{r}\left|\right|T_{i}\left|\right|k_{0}{)}^{a_{ir}}$ and $g^{a_{ir}},$ which are used by RSUs to classify the ciphertexts.

*Message Signing (MS)*: $V_{i}$ computes the signature $\sigma_{i}$ of $M_{i},$ where $\sigma_{i}={\left(H_{1}\left(M_{i}\right)\right)}^{s_{i}}$ [32]. After that, $V_{i}$ sends $\left(M_{i},\sigma_{i}\right)$ to RSU $R_{j}$.

### 4.4. Data Aggregation at RSUs

After RSU $R_{j}$ receives the messages $\left(M_{1},\sigma_{1}\right),\left(M_{2},\sigma_{2}\right),...,$ $(M_{n},\sigma_{n}),$ $R_{j}$ verifies $\left(\sigma_{1},\sigma_{2},...,\sigma_{n}\right)$ by executing the following ***Message Verification*** algorithm. Then, $R_{j}$ classifies $M_{11},...,M_{1\theta_{1}},$ $M_{21},$ ...$,M_{2\theta_{2}}$, ..., $M_{n1},...,M_{n\theta_{n}}$ according to the areas by running the ***Data Classification*** algorithm. Note that $M_{1}$= ($M_{11},M_{12},...,$ $M_{1\theta_{1}},T_{i},PID_{i}$),..., $M_{n}$= ($M_{n1},M_{n2},...,$ $M_{n\theta_{n}},T_{n},PID_{n}$). Finally, $R_{j}$ executes the ***Data Aggregation*** algorithm.

*Message Verification (MV)*. Firstly, $R_{j}$ will check if $PID_{1},...,PID_{n}$ are all registered in the Public key list; for any $PID_{i},$ if it is not listed in the Public key list, $R_{j}$ will not use $M_{i}$ to generate aggregation results. Assume $\left(PID_{1},PID_{2},...,PID_{\delta }\right)$ are included in the Public key list. $R_{j}$ further checks if $\hat{e}\left(g,\sigma_{i}\right)=\hat{e}\left(g^{s_{i}},H_{1}\left(M_{i}\right)\right)$ holds where i∈[1,δ]. If so, then $M_{i}$ is a valid message. Here, we say a message is valid if it is generated by a legitimate vehicle.

*Data Classification (DC)*: Assume $\left(M_{1},M_{2},...,M_{\delta }\right)$ are valid messages. When $R_{j}$ wants to check if $m_{i\theta_{i}}$ and $m_{l\theta_{l}}$ are collected in the same area and during the same period, where $M_{i\theta_{i}}=<H_{1}(Area_{\theta_{i}}\left|\right|T_{i}\left|\right|k_{0}{)}^{a_{i\theta_{i}}},g^{a_{i\theta_{i}}},C_{i\theta_{i}}>$ and $M_{l\theta_{l}}=<H_{1}(Area_{\theta_{l}}\left|\right|T_{l}\left|\right|k_{0}{)}^{a_{l\theta_{l}}},g^{a_{l\theta_{l}}},C_{l\theta_{l}}>$, $R_{j}$ will firstly recover $T_{i}$ and $T_{l}$ to check if $T_{i}=T_{l}$ holds. If $T_{i}=T_{l}$ is satisfied, $R_{j}$ will verify if the following equality holds

$$\hat{e}(H_{1}(Area_{\theta_{i}}\left|\right|T_{i}\left|\right|k_{0}{)}^{a_{i\theta_{i}}},g^{a_{l\theta_{l}}})\overset{?}{=}\hat{e}(H_{1}(Area_{\theta_{l}}\left|\right|T_{l}\left|\right|k_{0}{)}^{a_{l\theta_{l}}},g^{a_{i\theta_{i}}}).$$

If so, we have $Area_{\theta_{i}}=Area_{\theta_{l}}$. That is, $m_{i\theta_{i}}$ and $m_{l\theta_{l}}$ are collected in the same area.

***Data Accuracy***. We can see that the scheme achieves data accuracy, since each aggregation result maps to a specific area and time period. Specifically, (1) in Data Classification phase, RSUs classify the data according to the areas where and when the data was collected, which means only data collected in the same area and during the same time period will be aggregated together; (2) the aggregation results are signed by RSUs then sent to SP. That is, the aggregation results are generated by real RSUs but not impersonated RSUs; and (3) all the sensing data are generated by registered vehicles. $R_{j}$ will verify if $M_{i}$ is valid by checking whether $PID_{i}$ is included in the Public key list (as shown in Table 1) and verifying $\sigma_{i}$ by using the public key corresponding to $PID_{i}$. If $PID_{i}$ is not on the list or the signature is not valid, $M_{i}$ will not be used any more.

*Data Aggregation (DA)*: Assume that $\left(m_{1r},m_{2r},...,m_{kr}\right)$ are collected in the same area, $Area_{r}$, and during the same period, $T_{l}$, where r∈{1,...,t}.

***1***: RSU $R_{j}$ aggregates $\left(C_{1r},C_{2r},...,C_{kr}\right)$ by computing $C_{r}=\prod_{i=1}^{k}C_{ir},$ and then we obtain $C_{r}={\left(p+1\right)}^{\sum_{i=1}^{k}{m_{ir}}^{2}}\times \eta^{\sum_{i=1}^{k}m_{ir}}\times \prod_{i=1}^{k}H_{2}\left(\hat{e}\left(H_{1}\left(PID_{i},T_{l}\right),g^{s_{i}r_{i}}\right)\right)\;\mathrm{mod}\;p^{2}.$
***2***: Let $B_{r}=\left(L_{j},PID_{1},PID_{2},...,PID_{k},T_{l},M_{1r},C_{r}\right).$ $R_{j}$ generates an ID-based signature $\sigma_{r}=\left(u_{r},v_{r}\right)$ [33] on $B_{r}$ by using its private key $S_{L_{j}}=H_{1}(L_{j}\left|\right|R_{j}{)}^{s},$ where $u_{r}=H_{1}(L_{j}\left|\right|R_{j}{)}^{a},$*a* is randomly chosen from $Z_{q}^{*},$ and $v_{r}={S_{L_{j}}}^{a+H(B_{r},u_{r})}$. Afterwards, $R_{j}$ sends $\left(B_{r},u_{r},v_{r}\right)$ to SP.

**Remark** **3.** $M_{1r}$ is included in $B_{r}$ where the format of $M_{1r}$ is $(H_{1}(Area_{r}\left|\right|T_{l}\left|\right|k_{0}{)}^{a_{1r}},g^{a_{1r}},C_{1r},T_{l},PID_{1})$. Thus, SP can recover $Area_{r}$ from $M_{1r}$ in the following Statistical Analysis phase by using $H_{1}(Area_{r}\left|\right|T_{l}\left|\right|k_{0}{)}^{a_{1r}}$ and $g^{a_{1r}}$. Since only the sensing data collected in the same area will be aggregated, SP can conclude that all sensing data are collected in$Area_{r}$.

### 4.5. Statistical Analysis at SP

After SP receives the messages $(B_{r},u_{r},v_{r}),$ SP will verify if $B_{r}$ is valid by performing the ***Data Verification*** algorithm. If $B_{r}$ is valid, SP will execute the ***Area Recovery*** algorithm to recover $Area_{r},$ and run the ***Data Decryption*** algorithm to decrypt $C_{r}$ and compute E(·) and Var(·) in $Area_{r}$.

*Data Verification (DV)*: After SP receives $(B_{r},u_{r},v_{r}),$ SP verifies if the following equality holds

$$\hat{e}\left(g,v_{r}\right)\overset{?}{=}\hat{e}(P_{pub},u_{r}\times H_{1}(L_{j}\left|\right|R_{j}{)}^{H(B_{r},u_{r})}).$$

If so, $\left(u_{r},v_{r}\right)$ is a valid signature of $B_{r}$. SP concludes that $B_{r}$ is generated by $R_{j}$.

*Area Recovery (RA)*: SP extracts $M_{1r}$ from $B_{r}$. According to $M_{1r}=(H_{1}(Area_{r}\left|\right|T_{l}\left|\right|k_{0}{)}^{a_{1r}},g^{a_{1r}},C_{1r}),$ for any $Area_{i},i\in \left\{1,...,t\right\},$ SP verifies if the following equality is satisfied

$$\hat{e}(H_{1}(Area_{r}\left|\right|T_{l}\left|\right|k_{0}{)}^{a_{1r}},g)\overset{?}{=}\hat{e}(H_{1}(Area_{i}\left|\right|T_{l}\left|\right|k_{0}),g^{a_{1r}}).$$

If, for some $Area_{i},$ the equality holds, then SP concludes that all the data aggregated in $B_{r}$ are collected in $Area_{i}$.

*Data Decryption (DD)*: SP computes statistical data E(·) and Var(·) by executing the following steps:

***1***: SP recovers $\left(g^{s_{1}r_{1}},g^{s_{2}r_{2}},...,g^{s_{k}r_{k}}\right)$ according to $(PID_{1},$ $PID_{2},...,$ $PID_{k})$.
***2***: SP computes
  $$D=\frac{C_{r}}{\prod_{i=1}^{k}H_{2}\left(\hat{e}\left(H_{1}\left(PID_{i},T_{l}\right),g^{s_{i}r_{i}}\right)\right)}\;\mathrm{mod}\;p^{2}$$
  and
  $$\bar{D}=D^{p}={\left({\left(p+1\right)}^{\sum_{i=1}^{k}{m_{ir}}^{2}}\times \eta^{\sum_{i=1}^{k}m_{ir}}\right)}^{p}={\left(\eta^{\sum_{i=1}^{k}m_{ir}}\right)}^{p}\;\mathrm{mod}\;p^{2}.$$
***3***: SP uses Pollard’s method to recover $\sum_{i=1}^{k}m_{ir}$ and calculates
  $$\hat{D}=\frac{D}{\eta^{\sum_{i=1}^{k}m_{ir}}}={\left(p+1\right)}^{\sum_{i=1}^{k}{m_{ir}}^{2}}.$$
  Because $m_{ir}$ is within a small plaintext space {0,1,2,...kΔ}, $\Sigma_{i=1}^{k}{\left(m_{ir}\right)}^{2}<p$. Therefore, we obtain
  D^=1+p×∑i=1kmir2+∑i=2∑i=1kmir2pi×∑i=1kmir2i=1+p×∑i=1kmir2modp2.
***4***: SP computes $\sum_{i=1}^{k}{m_{ir}}^{2}=\frac{\hat{D}-1}{p}$. According to $\sum_{i=1}^{k}m_{ir}$ and $\sum_{i=1}^{k}{m_{ir}}^{2},$ SP computes E(M) and Var(M) of variable *M* for $Area_{r}$ where
  $$E\left(M\right)=\frac{\sum_{i=1}^{k}m_{ir}}{k}$$
  and
  $$Var\left(M\right)=E\left(M^{2}\right)-{\left(E\left(M\right)\right)}^{2}=\frac{\hat{D}-1}{p\times k}-{\left(E\left(M\right)\right)}^{2}.$$

Thus, SP gets E(M) and Var(M) of variable *M* for $Area_{r}$ at time period $T_{l}$. Similarly, SP can compute E(M) and Var(M) in other areas.

## 5. Security Analysis

Following aforementioned security requirements, our analysis will focus on how the proposed PAVS scheme can achieve the vehicles’ privacy-preserving property.

Assume vehicle $V_{i}$ collects sensing data in different areas, submits ciphertexts to RSU $R_{j},$ and $R_{j}$ classifies and aggregates the messages and sends them to SP. We will show that the proposed PAVS scheme can resist *sensing data link attack* by showing that it achieves full privacy, which means that $R_{j}$ will not get any valuable information from vehicles. In order to prove the proposed scheme achieves full privacy property, we explore the game sequence [34,35] to show that $R_{j}$ cannot distinguish the messages $M_{i}$ generated by vehicle $V_{i}$ from random strings, where $M_{i}=\left(M_{i1},M_{i2},...,M_{i\theta_{i}},T_{i},PID_{i}\right)$, and

$$\left\{\begin{array}{ccc} M_{i1} & = & <H_{1}(Area_{1}\left|\right|T_{i}\left|\right|k_{0}{)}^{a_{i1}},g^{a_{i1}},C_{i1}>\\ M_{i2} & = & <H_{1}(Area_{2}\left|\right|T_{i}\left|\right|k_{0}{)}^{a_{i2}},g^{a_{i2}},C_{i2}>\\ ... & = & ...\\ M_{i\theta_{i}} & = & <H_{1}(Area_{\theta_{i}}\left|\right|T_{i}\left|\right|k_{0}{)}^{a_{i\theta_{i}}},g^{a_{i\theta_{i}}},C_{i\theta_{i}}>. \end{array}\right.$$

The game sequence is explored to prove that the scheme is secure. This is because game sequence is a useful tool in taming the complexity of security proofs that might otherwise become complicated as to be nearly impossible to verify [35]. In our security proof, the attack games are played between an RSU $R_{j}$ and a challenger. Both $R_{j}$ and the challenger are probabilistic processes. In the proof, Game 0 and Game 1 are constructed, where Game 0 is the original attack game. If $R_{j}$ cannot distinguish Game 0 and Game 1, we can conclude that it cannot distinguish the messages generated by a vehicle from random strings. The challenger generates private keys for *n* vehicles so that it can act as real vehicles.

**Game 1**. If $R_{j}$ submits $\left(T_{l},PID_{l}\right)$ to the challenger, the challenger will choose $\left(m_{l1},...,m_{l\theta_{l}}\right)$ randomly as sensing data, answer $R_{j}$’s query by normally executing the scheme, and return the messages generated by $V_{l}$ to $R_{j}$.

At some point, $R_{j}$ submits $\left(T_{i},PID_{i}\right)$ (where $T_{i}$ is not queried before. If $T_{i}$ has been queried, $R_{j}$ may verify if $M_{0}^{*}$ and $M_{1}^{*}$ are generated by real vehicles though executing *Data Classification* algorithm; however, $R_{j}$ still cannot get any valuable information). The challenger generates two messages $M_{0}^{*}$ and $M_{1}^{*}$ to $R_{j}$, where

$$\begin{array}{r} M_{0}^{*}=(<H_{1}(Area_{1}\left|\right|T_{i}\left|\right|k_{0}{)}^{a_{i1}},g^{a_{i1}},C_{i1}>,<H_{1}(Area_{2}\left|\right|T_{i}\left|\right|k_{0}{)}^{a_{i2}},g^{a_{i2}},C_{i2}>,...,\\ <H_{1}(Area_{\theta_{i}}\left|\right|T_{i}\left|\right|k_{0}{)}^{a_{i\theta_{i}}},g^{a_{i\theta_{i}}},C_{i\theta_{i}}>,T_{i},PID_{i})\\ M_{1}^{*}=\left(<\omega_{1},\omega_{1}^{\prime },C_{i1}>,<\omega_{2},\omega_{2}^{\prime },C_{i2}>,...,<\omega_{\theta_{i}},\omega_{\theta_{i}}^{\prime },C_{i\theta_{i}}>,T_{i},PID_{i}\right). \end{array}$$

Here, $\omega_{1},\omega_{1}^{\prime },\omega_{2},\omega_{2}^{\prime },...,\omega_{\theta_{i}},\omega_{\theta_{i}}^{\prime }$ are all random strings, and $C_{i1},...,C_{i\theta_{i}}$ are generated by performing the scheme normally. The challenger selects a random b∈{0,1} uniformly, and then sends $M_{b}^{*}$ to $R_{j}$. $R_{j}$ will return 0 if it thinks that the whole message is generated by a real vehicle $V_{i}$. Otherwise, $R_{j}$ returns 1. We say $R_{j}$ can win Game0 with advantage ${\mathrm{Adv}}_{G0}\left(R_{j}\right)$, where ${\mathrm{Adv}}_{G0}\left(R_{j}\right)=\left|2{\mathrm{Pr}}_{G0}\left[b=b^{\prime }\right]-1\right|$.

**Game 2**. When $R_{j}$ submits $\left(T_{l},PID_{l}\right)$ to the challenger, the challenger will choose $\left(m_{l1},...,m_{l\theta_{l}}\right)$ randomly as sensing data, answer $R_{j}$’s query by normally executing the scheme, and return the messages generated by $V_{l}$ to $R_{j}$.

At some time point, assume $R_{j}$ queries on $\left(T_{i},PID_{i}\right)$ (where $T_{i}$ or $PID_{i}$ is not queried before). The challenger chooses two messages $M_{0}^{*}$ and $M_{1}^{*}$ to $R_{j}$, where

$$\begin{array}{c} M_{0}^{*}=\left(<\beta_{1},\beta_{1}^{\prime },C_{i1}>,<\beta_{2},\beta_{2}^{\prime },C_{i2}>,...,<\beta_{\theta_{i}},\beta_{\theta_{i}}^{\prime },C_{i\theta_{i}}>,T_{i},PID_{i}\right),\\ M_{1}^{*}=\left(<\alpha_{1},\alpha_{1}^{\prime },\alpha_{1}^{\prime \prime }>,<\alpha_{2},\alpha_{2}^{\prime },\alpha_{2}^{\prime \prime }>,...,<\alpha_{\theta_{i}},\alpha_{\theta_{i}}^{\prime },\alpha_{\theta_{i}}^{\prime \prime }>,T_{i},PID_{i}\right). \end{array}$$

Here, $\alpha_{1},...,\alpha_{\theta_{i}},\alpha_{1}^{\prime },...,\alpha_{\theta_{i}}^{\prime },\alpha_{1}^{\prime \prime },...,\alpha_{\theta_{i}}^{\prime \prime },\beta_{1},...,\beta_{\theta_{i}},$ $\beta_{1}^{\prime },...,$ $\beta_{\theta_{i}}^{\prime }$ are all random strings. The challenger selects a random b∈{0,1} uniformly and then sends $M_{b}^{*}$ to $R_{j}$. $R_{j}$ will return 0 if it thinks that the messages include some information generated by real vehicle $V_{i}$. Otherwise, $R_{j}$ will return 1. We say $R_{j}$ can win Game1 with advantage ${\mathrm{Adv}}_{G1}\left(R_{j}\right)$, where ${\mathrm{Adv}}_{G1}\left(R_{j}\right)=\left|2{\mathrm{Pr}}_{G1}\left[b=b^{\prime }\right]-1\right|$.

If the advantage with which $R_{j}$ wins Game0 and Game1 is both negligible, we can conclude that $R_{j}$ cannot get any valuable information.

We conclude that $R_{j}$ cannot distinguish the message generated by registered vehicles with random strings. Firstly, the advantage ${\mathrm{Adv}}_{G0}\left(R_{j}\right)$ with which $R_{j}$ wins Game 0 is negligible. That is, $R_{j}$ cannot distinguish ($H_{1}(Area_{k}\left|\right|T_{i}\left|\right|k_{0}{)}^{a_{ik}},g^{a_{ik}}$) from $(\omega_{k},\omega_{k}^{\prime }$). Let $h=g^{a_{ik}}$. Then, $H_{1}(Area_{k}\left|\right|T_{i}\left|\right|k_{0})$ can be denoted as $h^{b_{k}}$ for some unknown $b_{k}$. Similarly, $\omega_{k}$ can be denoted as $\left({\omega_{k}}^{\prime c_{k}}\right)$ for some unknown $c_{k}$. Since *h*, ${\omega_{k}}^{\prime }$, $b_{k}$, $c_{k}$ are all random elements, $R_{j}$ cannot distinguish ($h^{b_{k}},h$) from $\left({\omega_{k}}^{\prime c_{k}},\omega_{k}^{\prime }\right)$.

Secondly, the advantage ${\mathrm{Adv}}_{G1}\left(R_{j}\right)$ with which $R_{j}$ wins Game 1 is negligible. Assume that the challenge is to break a DBDH problem instance, i.e., to distinguish $c_{0}$ and $c_{1}$ given $g^{x}$, $g^{y}$, $g^{z},$ where $c_{0}=\hat{e}{\left(g,g\right)}^{xyz}$, $x,y,z\in Z_{q}^{*}$ and $c_{1}$ is a random element in $G_{T}$.

The challenger sets $V_{i}$’s public key $g^{s_{i}}$ as $g^{y}$, and $g^{r_{i}}$ as $g^{z}$. In Game1, $H_{1}$ is treated as a random oracle [36]. The output of $H_{1}\left(T_{i},PID_{i}\right)$ is set as $g^{x}$. Specifically, the challenger will generate $<C_{i1},...,C_{i\theta_{i}}>$ as follows:

$$\begin{array}{l} <C_{i1},...,C_{i\theta_{i}}>=<{\left(p+1\right)}^{{m{}_{i1}}^{2}}\times \eta^{m{}_{i1}}\times H_{2}\left(c_{0}\right),...,{\left(p+1\right)}^{{m{}_{i\theta_{i}}}^{2}}\times \eta^{m{}_{i\theta_{i}}}\times H_{2}\left(c_{0}\right)>,\\ <\alpha_{1}^{\prime \prime },...,\alpha_{\theta_{i}}^{\prime \prime },>=<{\left(p+1\right)}^{{m{}_{i1}}^{2}}\times \eta^{m{}_{i1}}\times H_{2}\left(c_{1}\right),...,{\left(p+1\right)}^{{m{}_{i\theta_{i}}}^{2}}\times \eta^{m{}_{i\theta_{i}}}\times H_{2}\left(c_{1}\right)>. \end{array}$$

$R_{j}$ returns a bit $b^{\prime }$ and guesses that $M_{b^{\prime }}$ is generated by vehicle $V_{i}$. If $R_{j}$ can distinguish a valid ciphertext from a random string with a non-negligible advantage *ε*, then the challenger can break the DBDH assumption with non-negligible advantage.

Therefore, we can conclude that $R_{j}$ cannot get any valuable information from the messages generated by registered vehicles. Thus, the proposed PAVS scheme captures full privacy, and can resist a *sensing data link attack*.

## 6. Performance Evaluation

In this section, we evaluate the performance of the proposed PAVS scheme in terms of computational cost, communication cost, and storage cost. In order to ease the presentation, we give the corresponding notations in Table 7.

### 6.1. Theoretical Analysis

According to the proposed PAVS scheme, the computational cost, communication cost and storage cost at vehicle side, RSU side, and SP side will be analyzed in this section.

#### 6.1.1. Computational Cost

In the proposed PAVS scheme, a vehicle needs to encrypt each piece of sensing data. Additionally, the vehicle will generate a signature. Since the vehicle can choose a generic signature to sign the messages, the performance of the signature is not analyzed here. For vehicle $V_{i}$ to encrypt each piece of sensing data, it needs to perform a pair operation, five exponentiations, and three multiplication operations.

If RSU $R_{j}$ receives $n_{dr}$ encrypted sensing data collected from $n_{a}$ different areas, it will classify the data. Assume $m_{c}$ and $m_{d}$ are collected in the same area. For any message $m_{e}$, in order to verify if $m_{e}$ is collected in the same area with $m_{c}$ and $m_{d}$, if $Area_{c}=Area_{e}$ has been verified, it is not necessary to verify if $Area_{d}=Area_{e}$ holds. Therefore, $R_{j}$ will execute at most $O(n_{dr}n_{a})$ pair operations to achieve data classification. For *k* ciphertexts, $R_{j}$ aggregates them by executing (k−1) multiplication operations.

Assume SP receives $n_{ds}$ aggregation results and all the sensing data are collected in $n_{a}$ areas. SP will execute at most $O(n_{ds}n_{a})$ pair operations to recover the areas. SP executes *k* multiplication operations and two exponentiation operations to compute $\bar{D}$. In addition, SP needs to use Pollard’s method to recover $\sum_{i=1}^{k}m_{ir}$ and performs one multiplication operation to calculate $\hat{D}$.

#### 6.1.2. Communication Cost

In the system initialization phase, TA will send long-term secrets to vehicles, RSUs and SP. After vehicles encrypt the sensing data, they will transfer the ciphertexts and a signature to RSUs. After that, RSUs will send messages to SP. The corresponding communication cost is listed in Table 8.

#### 6.1.3. Storage Cost

The storage cost is related to the phase of system initialization. The storage overhead in TA is $n_{r}S_{g}+\left(3+2n_{v}\right)S_{q}$+ 2$n_{v}S_{g}$ + $tS_{a}$+$n_{v}S_{id}$, where $n_{r}S_{g}+\left(3+2n_{v}\right)S_{q}$ is the cost to store long-term secrets for vehicles, RSUs, SP and TA itself, and 2$n_{v}S_{g}$ + $tS_{a}$+$n_{v}S_{id}$ is the cost to store the public lists. The storage overhead at vehicle $V_{i}$ is 2$S_{q}$+$S_{id}$, at RSU is $S_{g}$, and at SP is $n_{v}$($S_{id}$+$S_{g}$) +$S_{q}$ as shown in Table 9.

### 6.2. Experimental Simulation

#### 6.2.1. Implementation and Experimental Settings

The performance of PAVS is independent from the security parameters and the number of hash functions. Accordingly, Table 10 shows the parameter settings. The experiment is run on a test machine with Intel(R) Core(TM) I5-4200u 1.6 GHz four-core processor, 8 GB RAM, and a Windows 8 platform based on a Java Pairing-Based library [37].

#### 6.2.2. Computational Costs on the Vehicle Side

**Figure 2:**
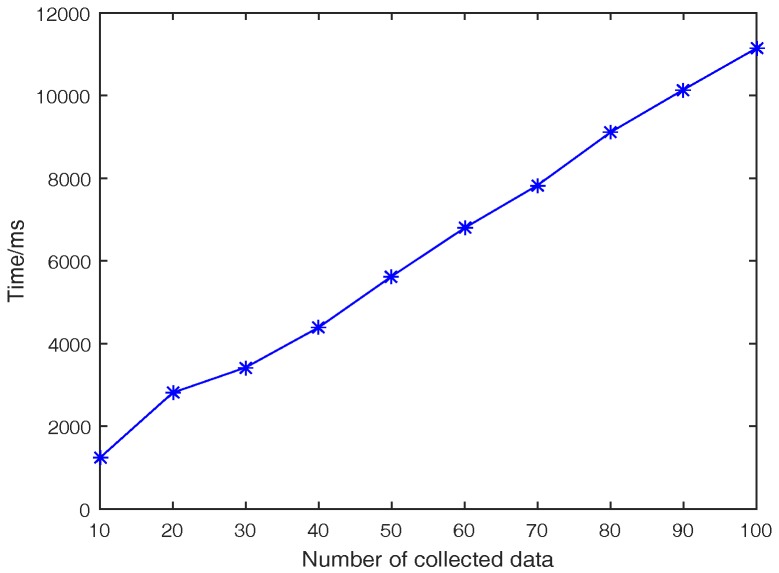
Computational costs on the vehicle side.

For a vehicle $V_{i}$, it needs to encrypt the sensing data, generate $M_{i}$ and sign $M_{i}$. Thus, in the experiments, the computational costs of $V_{i}$ are simulated by the total runtime including encryption, signature generation, and message generation algorithms on the vehicle side. On the vehicle side, the amount of sensing data $n_{dv}$ varies from 10 to 100. The change tendency of the computational cost on the vehicle side is shown in Figure 2. We can see that the computational cost is 1.235 s if $n_{dv}$ is 10, and 11.141 s when $n_{dv}$ equals 100. Therefore, the algorithms for vehicles are efficient enough.

#### 6.2.3. RSU’s Computational Cost

**Figure 3:**
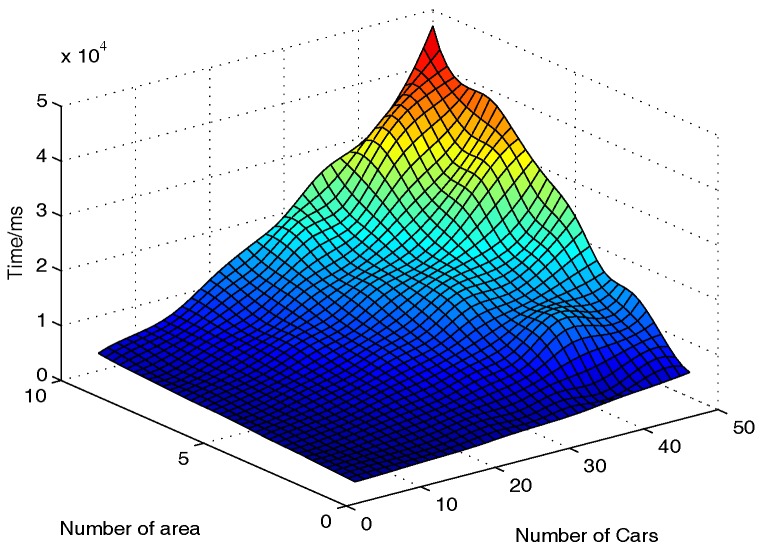
Computational costs on the RSU side.

On the RSU side, the RSUs need to verify if the received messages are valid, classify the messages and aggregate the messages. Therefore, the computational cost of an RSU is measured by the total runtime including Message Verification, Data Classification, and Data Aggregation algorithms. According to the proposed PAVS, the performance of data classification is not only related to the number of messages received by RSUs, but is also related to the number of areas which the vehicles pass by. That is, with different numbers of vehicles and areas, the computational cost of RSU will be different. Thus, we set the number of vehicles $n_{v}$ as {5, 10,..., 50} and the number of areas the vehicles pass by $n_{a}$ as {1, 2,..., 10}. As shown in Figure 3, although the increase of $n_{v}$ and $n_{a}$ leads to the increase in computational costs of RSU, the maximum running time is less than 48 s. Therefore, PAVS is efficient when computing on the RSU side, since the computation is not necessary to be in real time.

#### 6.2.4. SP’s Computational Cost

On the SP side, SP will verify if $B_{r}$ is valid, recover $Area_{r}$, and decrypt $C_{r}$. Therefore, the computational cost on the SP side is measured by the total runtime including the Data Verification algorithm, Area Recovery algorithm, and Data Decryption algorithm.

**Figure 4:**
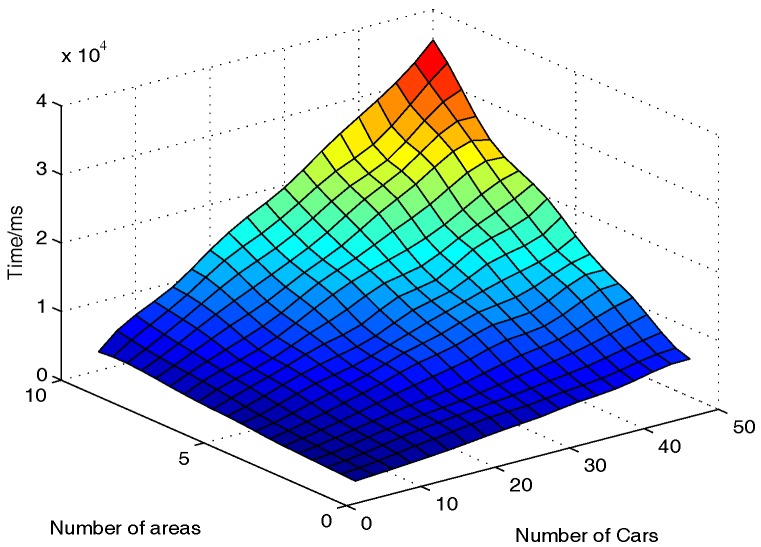
Computational costs on the SP side.

On the SP side, the number of vehicles $n_{v}$ and the number of areas $n_{a}$ which the vehicles pass by are still two core parameters. Accordingly, $n_{v}$ is chosen from 5 to 50 and $n_{a}$ is chosen from 1 to 10 to measure the computational overhead of different situations. The results are shown in Figure 4. Despite the fact that $n_{v}$ and $n_{a}$ increase, the running time of SP to get E(·) and Var(·) is less than 36 s, which is also acceptable.

### 6.3. Scalability

Assume SP has received the aggregation results $C_{1},C_{2},...,C_{n}$ of $Area_{1},Area_{2},...,Area_{n}$, respectively. If SP wants to get the statistical data E(·) and Var(·) of a larger area which includes some areas of $Area_{1},Area_{2},...,Area_{n}$, SP can still compute the new E(·) and Var(·) without re-executing the whole scheme.

**Figure 5:**
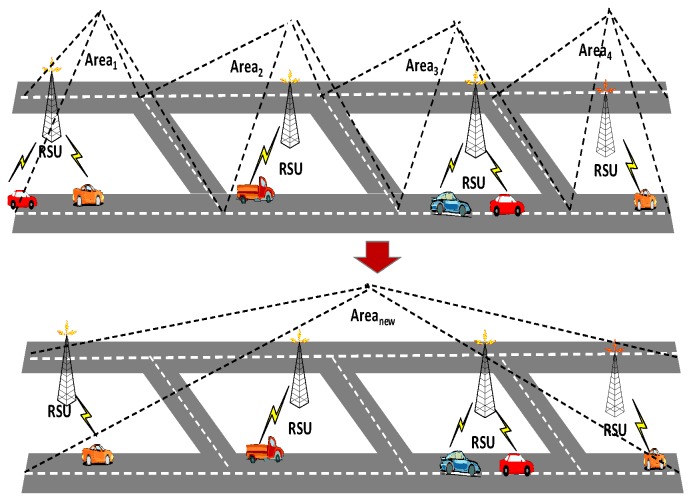
Area combining.

For instance, SP can get E(·) and Var(·) of a larger area which consists of $Area_{1},Area_{2},Area_{3},$ and $Area_{4}$ (as shown in Figure 5), as long as SP further aggregates $C_{1},C_{2},C_{3},C_{4}$ and then executes Step 2, Step 3 and Step 4 of the *Data Decryption* algorithm.

## 7. Related Works

In this section, we will mainly explore some of the existing work about VSS, since we propose a privacy-preserving data aggregation scheme for VSS.

In Ref. [10], Hu et al. constructed a VSS to monitor the concentration of carbon dioxide (CO${}_{2}$) gas. The VSS can collect CO${}_{2}$ concentration in a large field. Then, the collected data are reported to a remote server. The authors monitored the CO${}_{2}$ concentration in Hsin-Chu city, Taiwan, and the data are displayed on a Google map. However, the authors did not consider security issues in their scheme.

In Ref. [38], the authors proposed deploying mobile agents to collect sensor data from some specific road segments. The mobile agent moves among vehicles and communicates with the neighbour vehicles via wireless broadcast which may not reach all the vehicles in the given segment. In order to solve the problem, they proposed an agent-based data collection scheme that can help achieve close to 100% data collection rate. Similarly, in order to enhance sensing coverage, Masutani [39] proposed a route control method. The simulation experiment shows that the sensing coverage can be enhanced significantly without increasing the number of sensing vehicles.

Different to Ref. [38,39], Zhang et al. [40] proposed the maximum coverage quality with a budget constraint problem. They proposed a new algorithm by selecting some of mobile users to maximize the coverage quality. The results of the simulation experiments showed that their algorithm achieved better performance compared with the random selection scheme.

Freschi et al. proposed a data aggregation method [41] to monitor the roughness of road surfaces. In addition, a series of data aggregation schemes [17,18,19,20] have been proposed. However, security issues are not considered in these studies. In Ref. [42], the proposed scheme achieves authentication and integrity of aggregation data by aggregating the data and the message authentication codes. In order to tolerate duplicate messages, they also presented a probabilistic data aggregation scheme. However, privacy-preservation is not considered in [42].

Wu et al. proposed a hybrid routing scheme in urban hybrid networks [43]. They firstly presented a location-based crowd sensing framework. Then, they constructed a routing switch mechanism by utilizing ad hoc solutions and RSU resources to guarantee quality of data dissemination. In Ref. [44], the authors proposed a broadcast protocol that can support dense and sparse traffic regimes.

Lee et al. [9] proposed MobEyes to support urban monitoring. For MobEyes, vehicle-local processing capabilities are utilized to extract features, and mobile agents move and collect summaries from mobile nodes. If the agents identify interest data, they will contact the involved vehicles. In Ref. [45], Lee et al. further described MobEyes. They introduced the analytic model for MobEyes performance, the effects of concurrent execution of multiple harvesting agents, the valuation network overhead, and so on. Similarly, the privacy issues are not referred to in their work.

## 8. Conclusions

In this paper, we have proposed PAVS—an efficient privacy-preserving data aggregation scheme for VSS. Compared with existing schemes, the proposed PAVS scheme has been identified to compute the statistical data from aggregated encryption data. To realize PAVS, we have designed concrete privacy-preserving data classification and privacy-preserving aggregation algorithms. Detailed analysis shows it can resist a *sensing data link attack* and hold data accuracy and scalability. PAVS’s efficiency has been evaluated with theoretical analysis and experiments. Through extensive performance evaluations, we have demonstrated that the proposed PAVS’s scheme is efficient on the SP/RSU/vehicle sides.

## Figures and Tables

**Table 1:** Public key list.

PID	PK
$PID_{1}$	$g^{s_{1}}$
$PID_{2}$	$g^{s_{2}}$
$PID_{3}$	$g^{s_{3}}$
	...
$PID_{\beta }$	$g^{s_{\beta }}$

**Table 2:** Area list.

Areas
Area${}_{1}$
Area${}_{2}$
Area${}_{3}$
...
Area${}_{t}$

**Table 3:** Private key list.

RSUs	Private key
$R_{1}$	$H_{1}(L_{1}\left|\right|R_{1}{)}^{s}$
$R_{2}$	$H_{1}(L_{2}\left|\right|R_{2}{)}^{s}$
$R_{3}$	$H_{1}(L_{3}\left|\right|R_{3}{)}^{s}$
...	...
$R_{\alpha }$	$H_{1}(L_{\alpha }\left|\right|R_{\alpha }{)}^{s}$

**Table 4:** R-value list-1.

PID	$R_{1}$
$PID_{1}$	$r_{1}$
$PID_{2}$	$r_{2}$
$PID_{3}$	$r_{3}$
...	...
$PID_{\beta }$	$r_{\beta }$

**Table 5:** R-value list-2.

PID	$R_{2}$
$PID_{1}$	$g^{r_{1}}$
$PID_{2}$	$g^{r_{2}}$
$PID_{3}$	$g^{r_{3}}$
...	...
$PID_{\beta }$	$g^{r_{\beta }}$

**Table 6:** A-key list.

PID	A- key
$PID_{1}$	$g^{s_{1}r_{1}}$
$PID_{2}$	$g^{s_{2}r_{2}}$
$PID_{3}$	$g^{s_{3}r_{2}}$
...	...
$PID_{\beta }$	$g^{s_{\beta }r_{\beta }}$

**Table 7:** Notations for storage and communication cost analysis.

Notation	Definition
$n_{v}$	Number of vehicles
$n_{a}$	Number of areas
$n_{r}$	Number of RSUs
$n_{dv}$	Number of collected data of vehicle *V*
$n_{cr}$	Number of collected data received by RSU *R*
$n_{ds}$	Number of aggregation results received by SP
$S_{id}$	Bit size of pseudo identity for vehicle
$S_{rsu}$	Bit size of label for RSU
$S_{t}$	Bit size of time tamp
$S_{a}$	Bit size of area name
$S_{q}$	Bit size of an element in $Z_{q}^{*}$
$S_{p^{2}}$	Bit size of an element in $Z_{p^{2}}^{*}$

**Table 8:** Communication cost.

Entity	Communication Cost
TA	$\left(n_{r}+n_{v}\right)S_{g}+2n_{v}S_{id}+\left(2n_{v}+1\right)S_{q}$
Vehicle	$S_{r}+n_{dr}S_{id}+S_{t}+2S_{\eta }+2S_{q}$
RSU	$n_{dr}\left(2S_{g}+S_{\eta }\right)+S_{t}+S_{id}+S_{g}$

**Table 9:** Storage cost.

Entity	Storage Cost
TA	$n_{r}S_{g}+\left(3+2n_{v}\right)S_{q}$ + 2$n_{v}S_{g}$ + $n_{a}S_{a}$ + $n_{v}S_{id}$
Vehicle	2$S_{q}$ + $S_{id}$
RSU	$S_{rsu}$ + $S_{g}$
SP	$n_{v}$($S_{id}$ + $S_{g}$) + $S_{q}$

**Table 10:** Parameter settings.

Parameter	|q|	$|p^{\prime }|$	|p|	*H*	$H_{1}$	$H_{2}$
Setting	160 bit	512 bit	513 bit	160 bit	160 bit	513 bit
